# Association between smoking status and suicidal ideation, planning, and attempts among adults in South Korea: a population-based cross-sectional survey

**DOI:** 10.3389/fpsyt.2025.1440792

**Published:** 2025-06-06

**Authors:** Jae Han Kim, Taesic Lee, Hyo Jin Park, Seon Mee Kim, Jin-Wook Kim

**Affiliations:** ^1^ Yonsei University College of Medicine, Severance Hospital, Yonsei University Health System, Seoul, Republic of Korea; ^2^ Department of Family Medicine, Yonsei University Wonju College of Medicine, Wonju, Republic of Korea; ^3^ Department of Family Medicine, Ewha Woman’s University Seoul Hospital, Ewha Woman’s University College of Medicine, Seoul, Republic of Korea; ^4^ Department of Family Medicine, Korea University Guro Hospital, Korea University College of Medicine, Seoul, Republic of Korea; ^5^ Department of Family Medicine, Korea University College of Medicine, Seoul, Republic of Korea; ^6^ Hippocrata Clinic, Seoul, Republic of Korea

**Keywords:** smoking, suicidal ideation, suicidal planning, suicide attempt, South Korea

## Abstract

**Introduction:**

Suicide is a significant global public health concern influenced by diverse factors. Smoking is associated with an increased risk of suicide-related behaviors, yet age- and sex-specific risks remain unclear. This study examined the association between smoking status and suicide-related behaviors, with specific estimates for different age and sex groups.

**Methods:**

This study investigated the association between smoking status (never-smoker, ex-smoker, and current smoker) and suicide-related behaviors (suicidal ideation, planning, and attempts). We extracted the data from the Korea National Health and Nutrition Examination Survey database between January 1st, 2015, and December 31st, 2021. A total of 32,837 participants were included in this study, with a mean (SD) age of 52.3 (0.09) years. Multivariable logistic regression was adjusted for age, sex, BMI, income level, education level, active physical activity, alcohol consumption, and comorbid conditions (perceived stress, perceived symptoms of depression, hypertension, diabetes mellitus, and dyslipidemia). We explored the potential dose-response relationship by stratifying the analysis based on participants’ smoking intensity, as measured by pack-years. We also performed the subgroup analysis for the following variables: age groups, sex, income level, alcohol consumption, active physical activity, perceived stress, and perceived symptoms of depression. Statistical significance was set at *P* < 0.05. Statistical analyses were done using R software, version 4.2.3.

**Results:**

Current smokers demonstrated significantly elevated risks for suicidal ideation (OR 2.022, 95% CI 1.913–2.137), planning (OR 2.138, 95% CI 1.930–2.368), and attempts (OR 2.082, 95% CI 1.942–2.232). Ex-smokers also exhibited increased risks for suicidal ideation (OR 1.553, 95% CI 1.467–1.644) and attempts (OR 1.567, 95% CI 1.458–1.684), though not for planning (OR 1.087, 95% CI 0.963–1.228). Our findings suggested the potential dose-dependent relationship. Notably, males aged 40–59 were found to be the most vulnerable group for suicide-related behaviors.

**Conclusion:**

This study underscores the significant association of smoking with increased risks of suicidal behaviors, particularly among current smokers. Males aged 40 to 59 emerged as a high-risk group. Findings emphasize the critical role of smoking cessation in suicide prevention, necessitating targeted interventions. Prospective studies should delve into causal pathways to inform effective prevention strategies.

## 1 Introduction

Suicide remains a major concern of global public health. Suicidal behaviors are influenced by the interplay between clinical, biological, psychological, and environmental factors (1). According to the 2021 Global Burden of Disease study, the global burden of suicide was revealed to be significant. In 2021, the age-standardized mortality rate from suicide was estimated to be 8.99 per 100,000 individuals (95% uncertainty interval [UI] 8.34 to 9.64) (2). Within Asia, the rates varied across regions, with Central Asia at 9.73 (95% UI 8.78 to 10.70), South Asia at 11.6 (95% UI 9.77 to 12.6), East Asia at 7.28 (95% UI 6.25 to 8.83), and Southeast Asia at 4.80 (95% UI 4.20 to 5.39) (2). Notably, South Korea showed a notable age-standardized mortality rate of 18.1 per 100,000 individuals, 95% UI 11.1 to 19.7 (2). The mortality rate from suicide varied among different regions, genders, and age groups, which suggests that targeted strategies for suicide prevention are required for vulnerable populations.

While diverse preventative strategies are available for suicide, such as universal, selective, indicated, and multicomponent interventions (1), identifying modifiable risk factors is crucial for more targeted intervention and efficient use of limited resources. Herein, an umbrella review summarized the individual-level risk factors for suicide mortality. Among them, smoking was found to be associated with a two-fold increase in risk for suicide mortality (relative risk 2.4, 95% confidence interval [CI] 2.1 to 2.8) (3). Several studies have provided reliable evidence that smoking affects serotonin function, which can affect depression, major psychiatric disorders, and suicidal behavior (4, 5). Previous studies have firmly established observational associations between smoking and suicide-related behaviors, including suicidal ideation, planning, attempts, and completed suicides (6, 7). A systematic review with meta-analyses that encompassed 63 studies reported that current smokers were associated with an elevated risk of suicidal ideation (odds ratio [OR] 2.05, 95% CI 1.53 to 2.58), suicide planning (OR 2.36, 95% CI 1.69 to 3.02), and suicide attempts (OR 2.84, 95% CI 1.49 to 4.19) (7). Nonetheless, to the best of our knowledge, the stratified associations across age and sex groups have not yet been elucidated using a large number of participants. Previous meta-analytic studies did not conduct subgroup analyses for these moderators, while the burden of suicide varied among different age and sex groups.

Herein, this study aimed to investigate the association between smoking status and suicide-related behaviors (ideation, planning, and attempts). We conducted an additional analysis to explore the possible dose-responsive relationship by examining the associations according to smoking intensity, as measured by pack-years. Findings from this study could provide valuable insights for healthcare professionals and policymakers regarding the comparative risk of suicide-related behaviors in relation to smoking across different sex and age groups. This could highlight vulnerable populations and facilitate more targeted strategies for suicide prevention.

## 2 Methods

The study was conducted according to the guidelines of the Declaration of Helsinki and was approved by the Institutional Review Board of Wonju Severance Christian Hospital, Wonju, Republic of Korea (CR324321). The participant consent was waived since we used deidentified and retrospective data.

### 2.1 Participants recruitment

The current study utilized the Korea National Health and Nutrition Examination Survey (KNHANES) data. The KNHANES is a population-based survey with a cross-sectional design that is stratified, multistage, and clustered based on age, sex, and geographic area of participants. The KNHANES contains participants’ health status, including physical and laboratory examinations and nutritional assessments. Using the KNHANES data, we enrolled participant candidates from January 1st, 2015, to December 31st, 2021. Among them, we specifically included those aged 20 and above to ensure that only the adult population was enrolled. Then, we excluded individuals from the dataset whose data could not be used in the analyses due to missing information.

### 2.2 Study design and variables

This study aimed to explore the association between smoking status and suicidal ideation, planning, and attempts. We also investigated each of these associations by considering participants’ smoking intensity, as measured by pack-years. Moreover, we provided age-stratified estimates for each investigated association.

The participants’ smoking status was categorized as follows: ‘never-smokers’, ‘ex-smokers’, and ‘current smokers.’ We also evaluated the smoking intensity by examining the total number of cigarette packs they smoked over their lifetime, which was categorized into three groups: less than 10 pack-years, 10 to 15 pack-years, and 15 or more pack-years.

The outcomes of interest were participants’ suicidal ideation, planning, and attempts during the investigated periods. Suicidal ideation was evaluated with the query, “Have you seriously considered suicide in the past year?” Suicidal planning was gauged with the inquiry, “Have you made detailed plans to commit suicide in the past year?” Suicide attempts were examined with the question, “Have you actually tried to commit suicide in the past year?”

As covariates, we included the following variables in this study: age, sex, body mass index (BMI), income level, education level, active physical activity, alcohol consumption status, and participant’s comorbid conditions (perceived stress, perceived symptoms of depression, hypertension, dyslipidemia, and type 2 diabetes mellitus). Age groups were categorized into seven brackets: 20-29, 30-39, 40-49, 50-59, 60-69, 70-79, and 80 and over. Income levels were divided into four groups based on quartile ratios, ranging from Q1 (lowest) to Q4 (highest). Education levels were classified into four groups: elementary school or below, middle school graduate, high school graduate, and college graduate or above. Active physical activity was split into two categories—yes or no—depending on whether individuals engaged in moderate-intensity physical activity for 150 minutes or more per week, or high-intensity physical activity for 75 minutes or more per week, or a combination of moderate and high-intensity activities (where 1 minute of high-intensity equals 2 minutes of moderate-intensity), totaling the equivalent time for each activity. Alcohol consumption status was delineated into two groups: heavy consumption (≥ 140g/week for males or ≥ 70g/week for females) and non-heavy consumption (< 140g/week for males and < 70g/week for females). Perceived stress was evaluated with the query, “How much stress do you usually experience in your daily life?” categorized as severe, moderate, mild, and none. Perceived symptoms of depression were assessed with the inquiry, “Have you experienced feeling consistently sad or hopeless for two weeks or more to the extent that it significantly affected your daily life in the past year?”, with responses recorded as either yes or no. Hypertension was identified through a blood pressure reading of ≥140/90 mmHg or by the query “Are you currently taking medication to control your blood pressure?” Dyslipidemia was defined by a total cholesterol level of 240 mg/dL or higher. Type-2 diabetes mellitus was determined by a fasting blood glucose level of ≥126 mg/dL.

### 2.3 Statistical analysis

To evaluate the differences in study variables among the three groups (never-smokers, ex-smokers, and current smokers), we conducted the analysis of variance (ANOVA) followed by posthoc analysis for continuous variables and χ² test for categorical variables. The Kolmogorov-Smirnov normality analysis was implemented for continuous variables. If the result indicated normality, it was conveyed as mean and standard error. As a main analysis, we examined the association between smoking status and the risk of suicidal ideation, planning, and attempts. We employed multivariable logistic regression analysis, using never-smokers as the reference group, to estimate the OR and corresponding 95% CI for both ex-smokers and current smokers. To adjust for potential confounders, we tested four models: Model 1 (crude analysis), Model 2 (adjusted for age and sex), Model 3 (adjusted for age, sex, BMI, income level, education level, active physical activity, and alcohol consumption), and Model 4 (adjusted for age, sex, BMI, income level, education level, active physical activity, alcohol consumption, and comorbid conditions [perceived stress, perceived symptoms of depression, hypertension, diabetes mellitus, and dyslipidemia]). We assessed the goodness of fit for each main model using the Nagelkerke R-squared value, an adjusted version of the Cox & Snell R-square (8). Nagelkerke R-squared value ranges from 0 to 1, and a higher value signifies a better model fit, which indicates that the included covariates better explain the variability in the risk of suicidal ideation, planning, and attempts.

We explored the potential dose-response relationship by stratifying the analysis based on participants’ smoking intensity (less than 10 pack-years, 10 to 15 pack-years, and 15 or more pack-years). We also performed the subgroup analysis for the following variables: age groups (20-29, 30-39, 40-49, 50-59, 60-69, 70-79, and 80 and over), sex (male vs. female), income level (Q1, Q2, Q3, and Q4), alcohol consumption (heavy consumption vs. non-heavy consumption), active physical activity (yes vs. no), perceived stress (severe, moderate, mild, and none), and perceived symptoms of depression (yes vs. no).

The threshold for statistical significance was set at *P* < 0.05, and all statistical analyses were two-sided. Statistical analyses were performed using R software, version 4.2.3.

## 3 Results

### 3.1 Participants characteristics

Among the identified participant candidates between January 1st, 2015, and December 31st, 2021, a total of 32,837 participants were included in this study. Participants had a mean (SD) age of 52.3 (0.09) years and comprised more females (18,968 [57.8%]) than males (13,869 [42.2%]). Out of the 32,837 participants, 20,225 (61.6%) were classified as never-smokers, 7,313 (22.3%) as ex-smokers, and 5299 (16.1%) as current smokers. Regarding suicide-related behaviors, 924 (2.8%) individuals displayed suicidal ideation, 499 (1.5%) demonstrated suicidal planning, and 183 (0.6%) attempted suicide. The detailed characteristics of this study participants are presented in **Table 1**.

**Table 1:** Participants characteristics.

Study variables	Total participants	Never-smoker	Ex-smoker	Current smoker	*P*	*Post-hoc* ^†^
***N***	32837	20225	7313	5299		
Age (yrs: mean, SD; *N*, %)	52.3 ± 0.09	52.2 ± 0.12	55.8 ± 0.19	47.8 ± 0.21	<0.001	2-1:3-1:3-2
20-29	3628 (11.0)	2370 (11.7)	483 (6.6)	775 (14.6)		
30-39	4816 (14.7)	2915 (14.4)	914 (12.5)	987 (18.6)		
40-49	5920 (18.0)	3575 (17.7)	1210 (16.5)	1135 (21.4)		
50-59	6266 (19.1)	3858 (19.1)	1313 (18.0)	1095 (20.7)		
60-69	6175 (18.8)	3727 (18.4)	1635 (22.4)	813 (15.3)		
70-79	4550 (13.9)	2827 (14.0)	1318 (18.0)	405 (7.6)		
80+	1482 (4.5)	953 (4.7)	440 (6.0)	89 (1.7)		
Sex (*N*, %)					<0.001	2-1:3-1
Male	13869 (42.2)	3337 (16.5)	6139 (83.9)	4393 (82.9)		
Female	18968 (57.8)	16888 (83.5)	1174 (16.1)	906 (17.1)		
BMI (mean, SD)	24 ± 0.02	23.7 ± 0.03	24.4 ± 0.04	24.3 ± 0.05	<0.001	2-1:3-1:3-2
Income level (*N*, %)					<0.001	3-1
Q1	8012 (24.4)	4629 (22.9)	1766 (24.1)	1617 (30.5)		
Q2	8231 (25.1)	5045 (24.9)	1795 (24.5)	1391 (26.3)		
Q3	8286 (25.2)	5189 (25.7)	1850 (25.3)	1247 (23.5)		
Q4	8308 (25.3)	5362 (26.5)	1902 (26.0)	1044 (19.7)		
Education level (*N*, %)					<0.001	2-1:3-1
Elementary school or below	6769 (20.6)	4759 (23.5)	1303 (17.8)	707 (13.3)		
Middle school graduate	3341 (10.2)	1943 (9.6)	835 (11.4)	563 (10.6)		
High school graduate	10482 (31.9)	5989 (29.6)	2330 (31.9)	2163 (40.8)		
College graduate or above	12245 (37.3)	7534 (37.3)	2845 (38.9)	1866 (35.2)		
Active physical activity (*N*, %)					0.071	2-1
Yes	14124 (43.0)	8625 (42.6)	3231 (44.2)	2268 (42.8)		
No	18713 (57.0)	11600 (57.4)	4082 (55.8)	3031 (57.2)		
Alcohol consumption (*N*, %)*					<0.001	2-1:3-1
Heavy consumption	27421 (83.5)	18667 (92.3)	5475 (74.9)	3279 (61.9)		
Non-heavy consumption	5416 (16.5)	1558 (7.7)	1838 (25.1)	2020 (38.1)		
Comorbid conditions (N, %)						
*Perceived stress*					<0.001	2-1:3-1
Severe	1520 (4.6)	876 (4.3)	277 (3.8)	367 (6.9)		
Moderate	7085 (21.6)	4276 (21.1)	1356 (18.5)	1453 (27.4)		
Mild	18657 (56.8)	11600 (57.4)	4282 (58.6)	2775 (52.4)		
None	5575 (17.0)	3473 (17.2)	1398 (19.1)	704 (13.3)		
*Perceived symptoms of depression*					<0.001	2-1
Yes	2278 (6.9)	1457 (7.2)	409 (5.6)	412 (7.8)		
No	30559 (93.1)	18768 (92.8)	6904 (94.4)	4887 (92.2)		
*Hypertension*					<0.001	2-1:3-1
Yes	7731 (23.5)	4646 (23.0)	2088 (28.6)	997 (18.8)		
No	25106 (76.5)	15579 (77.0)	5225 (71.4)	4302 (81.2)		
*Dyslipidemia*					<0.001	3-1
Yes	4798 (14.6)	3113 (15.4)	1139 (15.6)	546 (10.3)		
No	28039 (85.4)	17112 (84.6)	6174 (84.4)	4753 (89.7)		
*Diabetes mellitus*					<0.001	2-1:3-1
Yes	3360 (10.2)	1860 (9.2)	973 (13.3)	527 (9.9)		
No	29477 (89.8)	18365 (90.8)	6340 (86.7)	4772 (90.1)		
Smoking intensity (pack-years: mean, SD)	6.9 ± 0.08	–	17.3 ± 0.23	18.8 ± 0.21	<0.001	2-1:3-1:3-2
Outcomes of interest (*N*, %)						
Suicidal ideation	924 (2.8)	494 (2.4)	199 (2.7)	231 (4.4)	<0.001	
Suicidal planning	499 (1.5)	253 (1.3)	107 (1.5)	139 (2.6)	<0.001	
Suicidal attempt	183 (0.6)	91 (0.4)	29 (0.4)	63 (1.2)	<0.001	

BMI, body mass index; N, number of participants; SD, standard deviation.

†N_1_-N_2_ indicates that Group N_1_ is greater than Group N_2_ (Group 1 = never-smoker; Group 2 = ex-smoker; Group 3 = current smoker).

Definitions of included variables are described in Methods section.

### 3.2 Main analysis: association between smoking status and suicidal ideation, planning, and attempts

Among the models, the fully adjusted model (Model 4) demonstrated the highest Nagelkerke R-squared values for all outcomes of interest: 0.365 for suicidal ideation, 0.179 for suicidal planning, and 0.195 for suicidal attempts (**Table 2**). After fully adjusting for potential confounders, ex-smokers were found to be associated with a statistically significant higher risk of suicidal ideation (OR 1.553, 95% CI 1.467 to 1.644) and suicide attempts (OR 1.567, 95% CI 1.458 to 1.684) compared to never-smokers, while the association with suicidal planning was not significant (OR 1.087, 95% CI 0.963 to 1.228). For current smokers, associations with suicidal ideation (OR 2.022, 95% CI 1.913 to 2.137), suicidal planning (OR 2.138, 95% CI 1.930 to 2.368), and suicide attempts (OR 2.082, 95% CI 1.942 to 2.232) retained statistical significance even after adjustment for potential confounders (**Table 2**).

**Table 2:** Association between smoking status and suicide-related behaviors in the adult population.

	Smoking status			Nagelkerke R-squared values
	Never-smoker	Ex-smoker	Current smoker	
***N* (total participants)**	20225	7313	5299	
Suicidal ideation				
*N* (number of cases)	494	199	231	
Model 1 (OR, 95% CI)	1 (reference)	1.067 (1.022 to 1.114)	1.887 (1.817 to 1.960)	0.009
Model 2 (OR, 95% CI)	1 (reference)	1.867 (1.774 to 1.964)	3.580 (3.414 to 3.753)	0.026
Model 3 (OR, 95% CI)	1 (reference)	1.789 (1.700 to 1.882)	2.881 (2.744 to 3.025)	0.058
Model 4 (OR, 95% CI)	1 (reference)	1.553 (1.467 to 1.644)	2.022 (1.913 to 2.137)	0.365
Suicidal planning				
*N* (number of cases)	253	107	139	
Model 1 (OR, 95% CI)	1 (reference)	0.849 (0.763 to 0.945)	2.709 (2.504 to 2.931)	0.01
Model 2 (OR, 95% CI)	1 (reference)	1.564 (1.387 to 1.764)	5.004 (4.542 to 5.512)	0.025
Model 3 (OR, 95% CI)	1 (reference)	1.371 (1.215 to 1.547)	3.318 (3.003 to 3.667)	0.057
Model 4 (OR, 95% CI)	1 (reference)	1.087 (0.963 to 1.228)	2.138 (1.930 to 2.368)	0.179
Suicidal attempt				
*N* (number of cases)	91	29	63	
Model 1 (OR, 95% CI)	1 (reference)	1.209 (1.140 to 1.283)	2.103 (1.996 to 2.217)	0.019
Model 2 (OR, 95% CI)	1 (reference)	1.996 (1.859 to 2.143)	3.873 (3.626 to 4.138)	0.031
Model 3 (OR, 95% CI)	1 (reference)	1.876 (1.748 to 2.014)	3.043 (2.844 to 3.256)	0.077
Model 4 (OR, 95% CI)	1 (reference)	1.567 (1.458 to 1.684)	2.082 (1.942 to 2.232)	0.195

CI, confidence interval; OR, odds ratio.

Our multivariable logistic regression adjusted for the following potential confounders: Model 1 (crude analysis), Model 2 (age and sex), Model 3 (age, sex, income level, body mass index [BMI], education level, active physical activity, and alcohol consumption), and Model 4 (age, sex, BMI, income level, education level, active physical activity, alcohol consumption, comorbid conditions [perceived stress, perceived symptoms of depression, hypertension, diabetes mellitus, and dyslipidemia]).

### 3.3 Potential dose-responsive relationship per smoking intensity

Regarding suicidal ideation, fully adjusted effect sizes were found to increase in individuals with greater pack-years in both ex-smoker and current smokers: for ex-smoker (<10 pack-years: OR 1.390, 95% CI 1.298 to 1.488; 10 to 15 pack-years: OR 1.810, 95% CI 1.604 to 2.042; >15 pack-years: OR 1.896, 95% CI 1.744 to 2.060) and for current smoker (<10 pack-years: OR 1.946, 95% CI 1.818 to 2.084; 10 to 15 pack-years: OR 1.955, 95% CI 1.763 to 2.168; >15 pack-years: OR 2.255, 95% CI 2.096 to 2.427) (**Table 3**).

**Table 3:** Stratified analysis based on smoking intensity for the associations between smoking status and suicide-related behaviors.

Suicide-related behaviors	Smoking intensity (pack-years)	OR (95% CI)			
		Model 1	Model 2	Model 3	Model 4
Suicide-related behaviors	Non-smoker	1 (reference)	1 (reference)	1 (reference)	1 (reference)
	Ex-smoker				
	<10	0.992 (0.937 to 1.050)	1.686 (1.586 to 1.793)	1.635 (1.538 to 1.739)	1.390 (1.298 to 1.488)
	10 to 15	0.875 (0.790 to 0.969)	1.754 (1.573 to 1.955)	1.820 (1.631 to 2.030)	1.810 (1.604 to 2.042)
	>15	1.266 (1.191 to 1.345)	2.486 (2.308 to 2.678)	2.287 (2.122 to 2.465)	1.896 (1.744 to 2.060)
	Current smoker				
	<10	1.827 (1.733 to 1.926)	3.396 (3.202 to 3.601)	2.650 (2.496 to 2.814)	1.946 (1.818 to 2.084)
	10 to 15	1.842 (1.695 to 2.002)	3.672 (3.360 to 4.013)	3.129 (2.860 to 3.423)	1.955 (1.763 to 2.168)
	>15	1.957 (1.862 to 2.056)	3.998 (3.757 to 4.255)	3.297 (3.092 to 3.515)	2.255 (2.096 to 2.427)
Suicidal planning	Non-smoker	1 (reference)	1 (reference)	1 (reference)	1 (reference)
	Ex-smoker				
	<10	0.701 (0.601 to 0.817)	1.156 (0.986 to 1.355)	1.039 (0.886 to 1.219)	0.839 (0.713 to 0.986)
	10 to 15	0.987 (0.788 to 1.236)	2.274 (1.793 to 2.884)	2.197 (1.731 to 2.787)	1.704 (1.341 to 2.165)
	>15	1.005 (0.860 to 1.173)	2.541 (2.120 to 3.045)	1.983 (1.654 to 2.378)	1.393 (1.160 to 1.674)
	Current smoker				
	<10	2.745 (2.474 to 3.046)	4.438 (3.954 to 4.982)	2.875 (2.554 to 3.236)	1.986 (1.761 to 2.240)
	10 to 15	3.920 (3.417 to 4.497)	7.874 (6.773 to 9.153)	5.766 (4.950 to 6.716)	3.466 (2.959 to 4.059)
	>15	2.298 (2.065 to 2.557)	5.399 (4.726 to 6.169)	3.508 (3.058 to 4.025)	2.005 (1.740 to 2.311)
Suicidal attempt	Non-smoker	1 (reference)	1 (reference)	1 (reference)	1 (reference)
	Ex-smoker				
	<10	1.043 (0.962 to 1.131)	1.782 (1.634 to 1.943)	1.701 (1.560 to 1.855)	1.424 (1.304 to 1.556)
	10 to 15	1.136 (0.996 to 1.295)	2.222 (1.930 to 2.558)	2.263 (1.965 to 2.606)	1.942 (1.683 to 2.241)
	>15	1.487 (1.370 to 1.614)	2.790 (2.521 to 3.088)	2.501 (2.259 to 2.769)	1.955 (1.763 to 2.167)
	Current smoker				
	<10	1.746 (1.616 to 1.886)	3.326 (3.055 to 3.621)	2.553 (2.342 to 2.782)	1.825 (1.671 to 1.994)
	10 to 15	1.461 (1.280 to 1.667)	2.904 (2.528 to 3.336)	2.409 (2.095 to 2.769)	1.482 (1.284 to 1.711)
	>15	2.633 (2.470 to 2.806)	5.250 (4.833 to 5.703)	4.210 (3.868 to 4.583)	2.773 (2.540 to 3.026)10 to 15

CI, confidence interval; OR, odds ratio.

Our multivariable logistic regression adjusted for the following potential confounders: Model 1 (crude analysis), Model 2 (age and sex), Model 3 (age, sex, income level, body mass index [BMI], education level, active physical activity, and alcohol consumption), and Model 4 (age, sex, BMI, income level, education level, active physical activity, alcohol consumption, comorbid conditions [perceived stress, perceived symptoms of depression, hypertension, diabetes mellitus, and dyslipidemia]).

For suicidal planning, individuals with 10 to 15 pack-years had the largest effect sizes when fully adjusted for both ex-smokers (<10 pack-years: OR 0.839, 95% CI 0.713 to 0.986; 10 to 15 pack-years: OR 1.704, 95% CI 1.341 to 2.165; >15 pack-years: OR 1.393, 95% CI 1.160 to 1.674) and current smokers (<10 pack-years: OR 1.986, 95% CI 1.761 to 2.240; 10 to 15 pack-years: OR 3.466, 95% CI 2.959 to 4.059; >15 pack-years: OR 2.005, 95% CI 1.740 to 2.311) (**Table 3**).

For suicide attempts, after full adjustment, individuals with >15 pack-years exhibited the largest effect sizes in both ex-smokers (<10 pack-years: OR 1.424, 95% CI 1.304 to 1.556; 10 to 15 pack-years: OR 1.942, 95% CI 1.683 to 2.241; >15 pack-years: OR 1.955, 95% CI 1.763 to 2.167) and current smokers (<10 pack-years: OR 1.825, 95% CI 1.671 to 1.994; 10 to 15 pack-years: OR 1.482, 95% CI 1.284 to 1.711; >15 pack-years: OR 2.773, 95% CI 2.540 to 3.026) (**Table 3**).

### 3.4 Subgroup analysis

**Figure 1:**
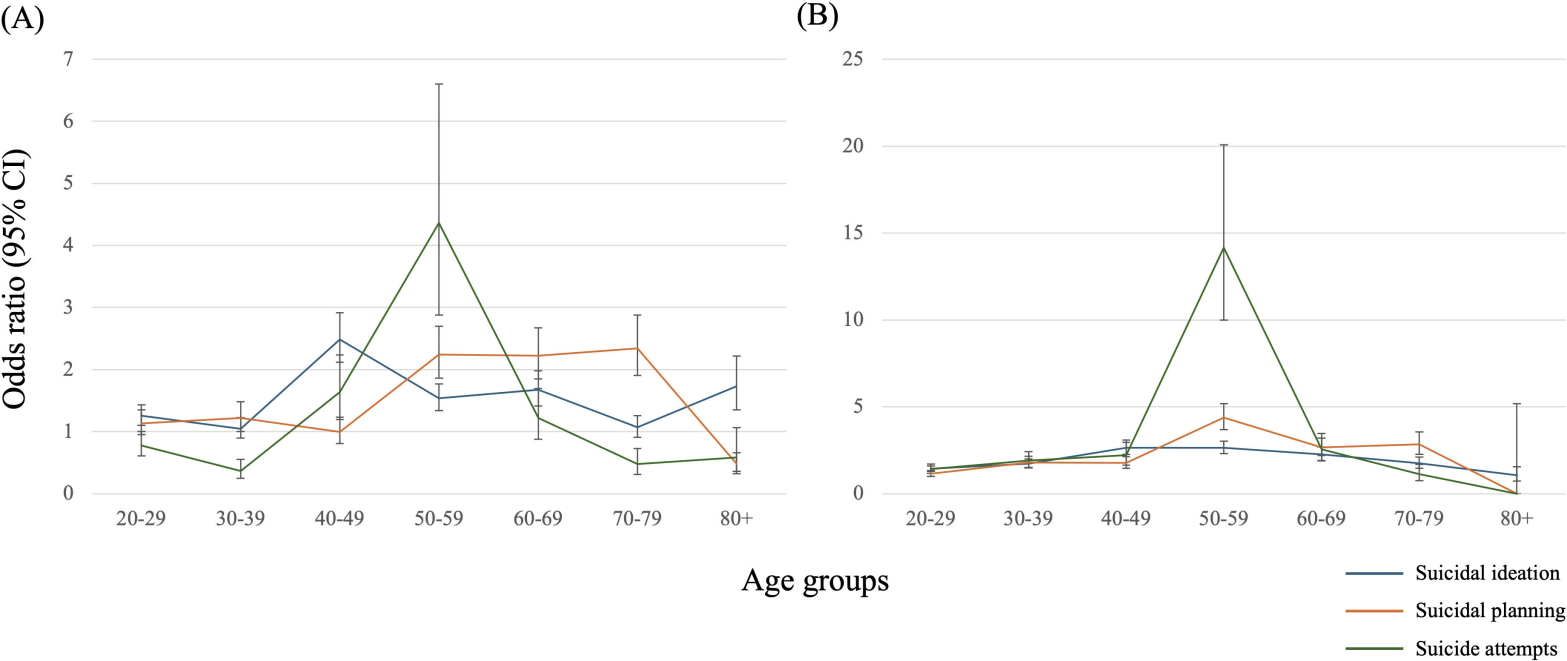
Age-stratified estimates on the association between smoking status and suicide-related behaviors. **(A)** ex-smoker, **(B)** current smoker.

Regarding age groups among ex-smokers, individuals aged 40 to 49 had the highest estimated odds for suicidal ideation (OR 2.487, 95% CI 2.120 to 2.918). The highest estimated odds for suicidal planning were found in those aged 50 to 79 (50 to 59: OR 2.240, 95% CI 1.860 to 2.699; 60 to 69: OR 2.224, 95% CI 1.849 to 2.675; 70 to 79: OR 2.342, 95% CI 1.907 to 2.876), while the largest estimate for suicide attempts was observed in the 50 to 59 age group (OR 4.360, 95% CI 2.880 to 6.602). Among current smokers, individuals aged 40 to 59 showed the largest estimates for suicidal ideation (40 to 49: OR 2.644, 95% CI 2.258 to 3.095; 50 to 59: OR 2.638, 95% CI 2.312 to 3.011). Notably, those aged 50 to 59 exhibited the greatest estimated odds for suicidal planning (OR 4.375, 95% CI 3.696 to 5.179) and suicide attempts (OR 14.159, 95% CI 9.985 to 20.077) (**Table 4**, **Figure 1**). In terms of sex, males exhibited a higher risk of suicidal ideation, planning, and attempts compared to females among both ex-smokers and current smokers (**Table 4**). Additionally, individuals with a Q2 income level, those who consumed alcohol heavily, engaged in no active physical activity, experienced higher perceived stress, and had perceived symptoms of depression showed higher estimates of these risks compared to other groups (**Table 4**).

**Table 4:** Subgroup analysis for the associations between smoking status and suicide-related behaviors.

Subgroup		Smoking status	Suicidal ideation	Suicidal planning	Suicidal attempt
			OR (95% CI)	OR (95% CI)	OR (95% CI)
Age	20-29	Never-smoker	1 (reference)	1 (reference)	1 (reference)
		Ex-smoker	1.255 (1.099 to 1.433)	1.134 (0.952 to 1.352)	0.779 (0.607 to 1.001)
		Current smoker	1.434 (1.282 to 1.603)	1.148 (0.988 to 1.335)	1.419 (1.179 to 1.708)
	30-39	Never-smoker	1 (reference)	1 (reference)	1 (reference)
		Ex-smoker	1.048 (0.897 to 1.225)	1.219 (1.003 to 1.481)	0.367 (0.244 to 0.551)
		Current smoker	1.730 (1.502 to 1.993)	1.796 (1.507 to 2.141)	1.895 (1.480 to 2.425)
	40-49	Never-smoker	1 (reference)	1 (reference)	1 (reference)
		Ex-smoker	2.487 (2.120 to 2.918)	0.997 (0.805 to 1.234)	1.634 (1.192 to 2.239)
		Current smoker	2.644 (2.258 to 3.095)	1.768 (1.467 to 2.130)	2.206 (1.643 to 2.962)
	50-59	Never-smoker	1 (reference)	1 (reference)	1 (reference)
		Ex-smoker	1.538 (1.337 to 1.770)	2.240 (1.860 to 2.699)	4.360 (2.880 to 6.602)
		Current smoker	2.638 (2.312 to 3.011)	4.375 (3.696 to 5.179)	14.159 (9.985 to 20.077)
	60-69	Never-smoker	1 (reference)	1 (reference)	1 (reference)
		Ex-smoker	1.672 (1.412 to 1.979)	2.224 (1.849 to 2.675)	1.220 (0.878 to 1.694)
		Current smoker	2.266 (1.908 to 2.691)	2.654 (2.201 to 3.202)	2.555 (1.886 to 3.461)
	70-79	Never-smoker	1 (reference)	1 (reference)	1 (reference)
		Ex-smoker	1.068 (0.907 to 1.257)	2.342 (1.907 to 2.876)	0.476 (0.312 to 0.725)
		Current smoker	1.746 (1.452 to 2.101)	2.839 (2.266 to 3.556)	1.115 (0.740 to 1.681)
	80+	Never-smoker	1 (reference)	1 (reference)	1 (reference)
		Ex-smoker	1.731 (1.351 to 2.217)	0.486 (0.359 to 0.658)	0.586 (0.323 to 1.063)
		Current smoker	1.057 (0.722 to 1.547)	0 (0 to inf.)	0 (0 to inf.)
Sex	Male	Never-smoker	1 (reference)	1 (reference)	1 (reference)
		Ex-smoker	1.531 (1.408 to 1.664)	1.454 (1.297 to 1.629)	1.239 (1.044 to 1.472)
		Current smoker	2.013 (1.865 to 2.172)	3.129 (2.864 to 3.419)	2.424 (2.134 to 2.754)
	Female	Never-smoker	1 (reference)	1 (reference)	1 (reference)
		Ex-smoker	1.281 (1.172 to 1.399)	1.001 (0.904 to 1.108)	0.531 (0.446 to 0.632)
		Current smoker	1.856 (1.703 to 2.023)	1.148 (1.038 to 1.270)	1.310 (1.128 to 1.522)
Income level	Q1	Never-smoker	1 (reference)	1 (reference)	1 (reference)
		Ex-smoker	1.306 (1.187 to 1.436)	1.520 (1.356 to 1.704)	0.826 (0.691 to 0.988)
		Current smoker	1.938 (1.776 to 2.115)	2.239 (2.015 to 2.488)	1.475 (1.267 to 1.717)
	Q2	Never-smoker	1 (reference)	1 (reference)	1 (reference)
		Ex-smoker	2.243 (2.009 to 2.504)	1.930 (1.691 to 2.201)	2.096 (1.624 to 2.707)
		Current smoker	2.213 (1.977 to 2.476)	1.909 (1.670 to 2.181)	5.436 (4.414 to 6.696)
	Q3	Never-smoker	1 (reference)	1 (reference)	1 (reference)
		Ex-smoker	1.568 (1.377 to 1.786)	1.124 (0.948 to 1.333)	1.488 (1.14 to 1.942)
		Current smoker	2.509 (2.214 to 2.843)	1.442 (1.218 to 1.708)	1.122 (0.85 to 1.480)
	Q4	Never-smoker	1 (reference)	1 (reference)	1 (reference)
		Ex-smoker	1.348 (1.176 to 1.546)	1.734 (1.401 to 2.145)	0 (0 to inf.)
		Current smoker	1.525 (1.317 to 1.766)	3.734 (3.018 to 4.619)	5.474 (4.131 to 7.255)
Alcohol consumption	Heavy consumption	Never-smoker	1 (reference)	1 (reference)	1 (reference)
		Ex-smoker	1.544 (1.448 to 1.646)	1.749 (1.615 to 1.894)	1.257 (1.002 to 1.577)
		Current smoker	2.302 (2.162 to 2.450)	2.270 (2.098 to 2.456)	2.007 (1.665 to 2.420)
	Non-heavy consumption	Never-smoker	1 (reference)	1 (reference)	1 (reference)
		Ex-smoker	1.269 (1.116 to 1.443)	0.880 (0.746 to 1.039)	0.895 (0.773 to 1.036)
		Current smoker	1.249 (1.110 to 1.406)	1.418 (1.226 to 1.640)	2.037 (1.808 to 2.296)
Active physical activity	Yes	Never-smoker	1 (reference)	1 (reference)	1 (reference)
		Ex-smoker	1.244 (1.137 to 1.361)	1.393 (1.250 to 1.553)	0.709 (0.583 to 0.861)
		Current smoker	2.074 (1.909 to 2.254)	1.613 (1.452 to 1.794)	2.040 (1.757 to 2.369)
	No	Never-smoker	1 (reference)	1 (reference)	1 (reference)
		Ex-smoker	1.855 (1.723 to 1.998)	1.697 (1.542 to 1.868)	1.486 (1.273 to 1.734)
		Current smoker	2.071 (1.922 to 2.231)	2.487 (2.267 to 2.728)	2.199 (1.914 to 2.526)
Perceived stress	Severe	Never-smoker	1 (reference)	1 (reference)	1 (reference)
		Ex-smoker	4.845 (3.549 to 6.615)	2.084 (1.507 to 2.883)	1.077 (0.868 to 1.337)
		Current smoker	8.920 (6.536 to 12.174)	1.661 (1.141 to 2.418)	2.396 (2.023 to 2.838)
	Moderate	Never-smoker	1 (reference)	1 (reference)	1 (reference)
		Ex-smoker	2.627 (2.370 to 2.911)	2.027 (1.778 to 2.311)	0.454 (0.349 to 0.590)
		Current smoker	2.449 (2.194 to 2.734)	1.353 (1.161 to 1.576)	1.691 (1.425 to 2.007)
	Mild	Never-smoker	1 (reference)	1 (reference)	1 (reference)
		Ex-smoker	1.084 (0.992 to 1.185)	1.459 (1.285 to 1.657)	1.896 (1.536 to 2.342)
		Current smoker	1.995 (1.843 to 2.160)	3.266 (2.924 to 3.648)	2.766 (2.261 to 3.384)
	None	Never-smoker	1 (reference)	1 (reference)	1 (reference)
		Ex-smoker	1.104 (0.974 to 1.252)	0.904 (0.787 to 1.039)	1.527 (0.730 to 3.195)
		Current smoker	1.237 (1.101 to 1.390)	1.405 (1.246 to 1.584)	0 (0 to inf.)
Perceived symptoms of depression	Yes	Never-smoker	1 (reference)	1 (reference)	1 (reference)
		Ex-smoker	1.619 (1.481 to 1.769)	1.947 (1.731 to 2.191)	1.442 (1.223 to 1.699)
		Current smoker	2.205 (2.018 to 2.410)	2.347 (2.101 to 2.622)	3.884 (3.388 to 4.452)
	No	Never-smoker	1 (reference)	1 (reference)	1 (reference)
		Ex-smoker	1.518 (1.411 to 1.635)	1.376 (1.256 to 1.507)	0.777 (0.648 to 0.932)
		Current smoker	1.940 (1.808 to 2.081)	1.955 (1.788 to 2.137)	1.042 (0.892 to 1.217)

CI, confidence interval; inf., infinite; OR, odds ratio.

Definitions of included variables are described in Methods section.

## 4 Discussion

This observational study investigated the association of smoking status with suicidal ideation, planning, and attempts, respectively, using a population-based cross-sectional survey of South Korea. After accounting for potential confounders, ex-smokers and current smokers were found to be associated with a higher risk of suicidal ideation, planning, and attempts compared to never-smokers, except that ex-smokers showed a non-significant result on suicidal planning. These findings were in line with prior studies reporting a significant association of current smoking with suicide that was not observed in ex-smokers (9). Of note, the effect sizes tended to be greater among current smokers than ex-smokers across all associations that were examined in the main analysis. Moreover, our findings suggested a potential dose-dependent relationship based on the individual’s smoking intensity. A Nepal study also demonstrated that smoking exhibited a dose-response relationship with suicide ideation (10).

The observational associations between smoking and suicide-related behaviors have been firmly established from previous studies, including meta-analyses (6, 7), reporting an approximately two-fold increased risk of suicide-related behaviors in ex-smokers or current smokers when compared to never-smokers. However, the evidence was primarily based on case-control or cross-sectional studies; specifically, a meta-analysis of prospective cohort studies identified only three studies for suicidal ideation and suicide attempts, respectively. Furthermore, previous evidence could not robustly determine the magnitude of the associations stratified by age and sex, despite these variables being important moderators. Herein, this study observed the association by participants’ age and sex, using the data from a population-based survey in South Korea. Our findings indicated that current smokers exhibited a similar two-fold increase in suicidal ideation, planning, and attempts, whereas ex-smokers demonstrated reduced effect sizes. Notably, our subgroup analyses identified the most vulnerable age group for each association. The results indicated that individuals aged 40 to 59 were associated with the highest risk for suicidal ideation, planning, and attempts among all age groups. Additionally, males were associated with a higher risk compared to females for all outcomes. The Japan Public Health Center-based Prospective Study demonstrated that smoking was a major predictor of suicide in middle-aged people with average ages ranging from 49 to 57 years old (11). Given these findings, we suggested that clinicians and policymakers should be alerted to the heightened risk of suicide-related behaviors among males aged 40 to 59.

Some previous studies suggested the potential causal relationship between smoking and suicide-related behaviors. Specifically, biological and behavioral mechanisms have been proposed. Regarding the biological mechanism, the current evidence indicates that smoking may lead to reduced serotonin levels and disruption of the hypothalamic-pituitary-adrenal (HPA) axis, both of which potentially impact an individual’s mood and increase the risk of suicide-related behaviors (4, 12, 13). The previous findings further corroborated this explanation that exposure to secondhand smoke was associated with suicide-related behaviors in certain populations, albeit with smaller effect sizes compared to direct smoking (14, 15). When considering both the reduction in serotonin levels and disruption of the HPA axis due to smoking were reported to have a proportional relationship with the amount of smoking (4, 12), our findings that 1) the magnitude of associations were greater in current smokers than ex-smokers and 2) observed potential dose-dependent relationship based on the individual’s smoking intensity may also support the possible biological link.

Nicotine represents a strong activator of the HPA axis, then, is related to an attenuated responsiveness of the HPA axis to psychological stress (13). The HPA alteration, also referred to as blunted axis activity, was demonstrated to increase risk for suicide attempt (16). In addition, most depressed patients have a dysregulation of corticotropin and cortisol secretory activity, as well as a tendency to show a high slope increase in the evening, when compared to the daytime (17, 18). An adolescent suicide prediction model suggested that depressive disorder is the main predictor of suicidal behaviors (19). In summary, smoking can be estimated to cause suicidal behavior directly or indirectly through HPA alteration.

However, recent studies suggest that behavioral mechanisms should also be addressed. Indeed, a previous study that utilized bidirectional Mendelian randomization and single nucleotide polymorphism analysis reported non-significant results (OR for lifetime smoking on suicidal ideation: 0.050; 95% CI -0.027 to 0.127; OR on suicide attempts: 0.053; 95% CI -0.003 to 0.110) (20). These previous findings implied that behavioral components might at least partially moderate the observed association between smoking and suicide-related behaviors. One possible hypothesis is that smoking status may indicate an individual’s impulsivity, which is also related to suicide-related behaviors (20, 21). This is further supported by our findings that observed effect sizes among current smokers were greater than those among ex-smokers (i.e., ex-smokers possibly had less impulsivity than current smokers). Meanwhile, the Mendelian randomization of smoking with depression (OR 1.00, 95% CI 0.95 to 1.05), anxiety (OR 1.02, 95% CI 0.97 to 1.07), and psychological distress (OR 1.02, 95% CI 0.98 to 1.06) also reported non-significance (22), refuting the previously suggested explanation that association between smoking and suicide-related behaviors was moderated by mental illnesses (5). However, the proportion of biological (e.g., nicotine-mediated) and behavioral (e.g., impulsivity-mediated) components for this association still needed to be investigated. With an effort to elucidate the underlying mechanisms, clinicians and policymakers should endeavor to promote smoking cessation, considering the evidence that smoking is a modifiable risk factor for not only mental illness, including suicide-related behaviors (3, 23, 24), but also numerous physical diseases (25–27).

Our findings have some limitations. First, identified cases of suicidal ideation, planning, and attempts may be underreported since the KNHANES data primarily relies on self-reporting, which implies that the odds in real-world practice could be higher. Second, the majority of data from KNHANES relied on self-reporting, implying that important variables such as smoking habits were inevitably susceptible to recall bias. Third, this study used the data with a population-based cross-sectional design, indicating that some confounding factors, such as the scale for participants’ impulsivity, could not be evaluated. However, we endeavored to include all accessible variables from the KNHANES database that could potentially influence the investigated associations. We also utilized multivariable logistic regression analysis to mitigate confounding effects and enhance the robustness of our findings. Fourth, since the associations in this study were derived from observational evidence, causality could not be established based on our findings. Further studies are warranted that employ prospective designs to investigate the moderators influencing the observed associations. Recently, albeit cross-sectional design, Mendelian randomization, which utilizes single-nucleotide polymorphisms as instrumental variables, has been employed to assess causal relationships between candidate features (20, 22).

Despite these limitations, this study re-affirmed the significant association between smoking and increased risk of suicidal ideation, planning, and attempts, with current smokers exhibiting stronger associations than ex-smokers. We further provided the stratified association by age and sex, of which the results indicated that males aged 40 to 59 should gain more attention. Our findings indicated a potential dose-dependent relationship between smoking and suicide-related behaviors. Despite limitations, including reliance on self-reported data, our results emphasized the importance of smoking cessation as a modifiable risk factor for suicide-related behaviors. Future studies should aim to elucidate the causality and consider prospective studies to better understand the complex interplay between smoking and suicide-related behaviors.

## Data Availability

The data analyzed in this study is subject to the following licenses/restrictions: Participant data used for this study cannot be publicly opened due to patient privacy concerns but can be shared by corresponding authors (J-WK: jwkim2011@naver.com) if a researcher provides a reasonable request. Requests to access these datasets should be directed to J-WK, jwkim2011@naver.com.
