# Supplementary material for: Association between smoking status and suicidal ideation, planning, and attempts among adults in South Korea: a population-based cross-sectional survey
Source: Front Psychiatry. 2025 Jun 6;16:1440792. doi: 10.3389/fpsyt.2025.1440792 (PMC12179204; doi:10.3389/fpsyt.2025.1440792)
Supplement: Supplementary file 1 [file Table1.docx]

**Table S1. Sequential Exclusion of Participants by Study Year**

| **Study year** | **Initial number of participants** | **Age criteria** | **Socioeconomic status** | **Life style** | **Medical diseases** | **Outcomes of interests** |
| --- | --- | --- | --- | --- | --- | --- |
| 2015 | 7380 | 5855 | 5499 | 4896 | 4890 | 4378 |
| 2016 | 8150 | 6315 | 6033 | 5700 | 5694 | 4854 |
| 2017 | 8127 | 6458 | 6098 | 5724 | 5719 | 5031 |
| 2018 | 7992 | 6424 | 6111 | 5836 | 5834 | 5128 |
| 2019 | 8110 | 6542 | 6165 | 5805 | 5803 | 5074 |
| 2020 | 7359 | 6072 | 5745 | 5265 | 5264 | 4093 |
| 2021 | 7090 | 5897 | 5494 | 5158 | 5154 | 4279 |
| Sum | 54208 | 43563 | 41145 | 38384 | 38358 | 32837 |
